# Spaceborne SAR Imaging Algorithm for Coherence Optimized

**DOI:** 10.1371/journal.pone.0148823

**Published:** 2016-02-12

**Authors:** Zhiwei Qiu, Jianping Yue, Xueqin Wang, Shun Yue

**Affiliations:** 1 School of Earth Science and Engineering, Hohai University, Nanjing 211100, China; 2 School of Surveying and Engineering, Henan University of Urban Construction, Pingdingshan 467036, China; Southwest University, CHINA

## Abstract

This paper proposes SAR imaging algorithm with largest coherence based on the existing SAR imaging algorithm. The basic idea of SAR imaging algorithm in imaging processing is that output signal can have maximum signal-to-noise ratio (SNR) by using the optimal imaging parameters. Traditional imaging algorithm can acquire the best focusing effect, but would bring the decoherence phenomenon in subsequent interference process. Algorithm proposed in this paper is that SAR echo adopts consistent imaging parameters in focusing processing. Although the SNR of the output signal is reduced slightly, their coherence is ensured greatly, and finally the interferogram with high quality is obtained. In this paper, two scenes of Envisat ASAR data in Zhangbei are employed to conduct experiment for this algorithm. Compared with the interferogram from the traditional algorithm, the results show that this algorithm is more suitable for SAR interferometry (InSAR) research and application.

## Introduction

In physics, it needs to have certain conditions for the stable interference generation. Only when the two columns of wave to meet have the same frequency, will there appear interference phenomenon and stable interference pattern be formed. The same theory can also be applied to synthetic aperture radar, including the single track double antenna pattern and single antenna repeat orbit mode. Although the radar transmitting microwave signals is homologous with the same frequency, the returned complex data sets are the record of echo signals from observed area. Hence, the coherence of two interference pairs would be reduced due to decoherence factors such as imaging posture and terrain features, and then the generated interferogram quality will be influenced. The generation of interference fringes with high quality in InSAR technology is an important prerequisite for obtaining the elevation and deformation with high accuracy. The coherence is a key index to evaluate the interferogram quality, which is that the carrier of the linear frequency modulation signal and reference signal maintain a constant phase difference, and will not change with time changing [1]. Correlation coefficient is used to measure the similarity between the pairs, which is defined as:

$$\gamma =\frac{\left|E[\mu_{1}\mu_{2}^{*}]\right|}{\sqrt{E[|\mu_{1}{|}^{2}]E[|\mu_{2}{|}^{2}]}}=|\gamma |e^{j\phi }$$ (1)

*μ*_1_ and *μ*_2_ represent the master and slave complex data respectively. * means complex conjugate, *E*[∙] represents mathematical expectation, |*γ*| is complex correlation coefficient, and *ϕ* is the phase of complex correlation coefficient. Correlation coefficient |*γ*| should be always less than 1, and the better the quality of interference fringes is, the higher the elevation accuracy is.

Correlation coefficient is standardized covariance function, which can reflect the degree of linear correlation between two signals. It is often used to measure the interferometric phase accuracy. Area with high coefficient also has a high similarity degree between two echoes, and then accurate distance can be acquired from the phase difference. Moreover, the place with low coherence also has low similarity degree between two echoes, the phase difference with low coherence doesn't completely represent the distance difference between echoes. It is difficult for interferometry to calculate the right results. In addition, coherence can reflect whether the terrain changes during the interval between the two illuminations, and it can also be used as an index of SAR image classification [2]. Ideally, the mathematical expectation in formula (1) can be obtained by calculating assembly average from interference patterns acquired under the same condition and same time. However, this method is difficult to carry out in fact. So, generally, it is assumed that is N pixels in the window have smooth random process. The assembly average of N pixels can be replaced by spatial mean, then the estimated value $\left|\hat{\gamma }\right|$ of coherence can be obtained by (represented by *γ* below) [3].

$$|\bar{\gamma }|=\frac{\sum_{n-1}^{N}\left|\mu_{1}^{\left(n\right)}\mu_{2}^{*(n)}\right|}{\sqrt{\sum_{n-1}^{N}|\mu_{1}^{\left(n\right)}{|}^{2}\sum_{n-1}^{N}|\mu_{2}^{\left(n\right)}{|}^{2}}}$$ (2)

It is well known that SAR is a kind of side-view imaging radar system, pulse compression technology is used to improve resolution of range direction, and the synthetic aperture technique is adopted to improve the azimuth resolution. The InSAR system coherence is affected by both this special focusing method and the interferometry mechanism. Now, the major factors [4–5] to cause the decorrelationare summarized as follows:

### (1) Decorrelation by baseline [6]

**Fig 1:**
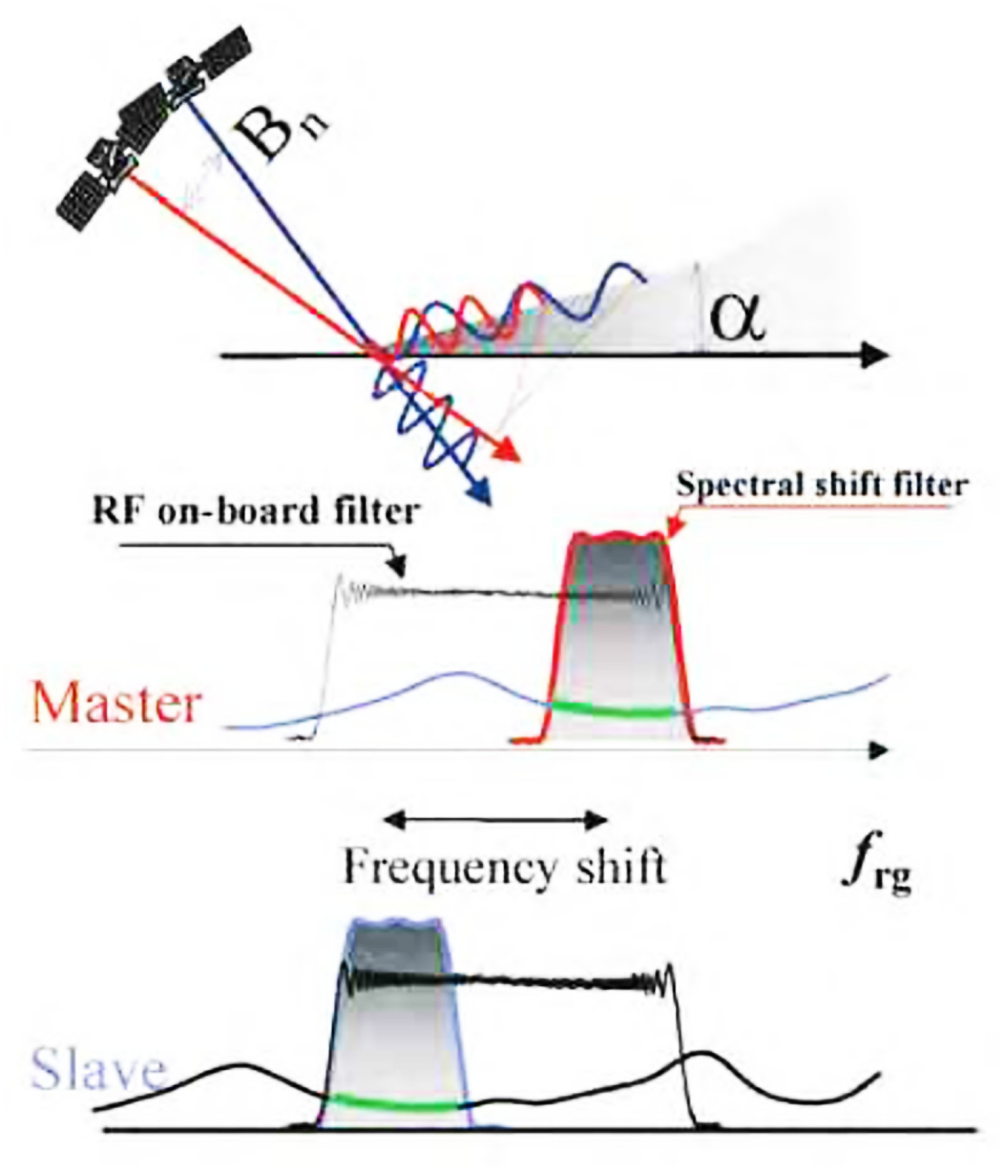
Decorrelation by baseline.

When the baseline is shorter, interference method is used to get higher accuracy. With the baseline increased, spectrum of range direction will shift, and the noise also will increase in interference process (see Fig 1). These not only decrease the accuracy of measurement, but also result in the decorrelation phenomenon of two pairs. The relation of frequency spectrum offset in range direction with baseline, radar looking angle, and so on is shown as follows.

$$\Delta f=-f_{0}\frac{B}{r_{0}\text{tan}(\theta -\alpha )}$$ (3)

In formula (3), *B* represents the length of spatial baseline, *r*_0_ is slant distance between the radar and ground, *f*_0_ represents center frequency of radar system, *θ* represents radar looking angle, *α* is the terrain slope angle. But the baseline can't reduce infinitely, interference fringe will become very sparse if baseline is decreased, and many details of the terrain information will be also lost. Prior filtering always is used to improve the correlation between the pairs along range direction before interferogram generation, but spectrum information will be abandoned after filtering process.

### (2) Frequency spectrum inconformity of azimuth

When using interference mode of single antenna repeat orbit, the antenna pointing will change due to different radar squint angles *φ*. Under ideal condition, the antenna beam should be perpendicular to the orbital plane, but for spaceborne SAR system, in addition to the own attitude pitching of platform, the earth rotation can also cause frequency shift. The relation of doppler centroid *f*_*dc*_, squint angle *φ*, and the correlation coefficient |*γ*_*a*_| is shown as follows [7].

$$f_{DC}=2v\cdot \text{sin}\varphi /\lambda$$ (4)

$$|\gamma_{a}|=1-\frac{2\text{sin}\theta |\Delta \varphi |\delta_{a}}{\lambda }$$ (5)

In formulas (4) and (5), Δ*φ* represents the difference of squint angle between two observations, *δ*_*a*_ is the azimuth resolution, and *λ* is radar wavelength. The formula (4) take a derivative with respect to the radar squint angle, then the result is substituted into formula (5).

The relationship between coherence the doppler center frequency is expressed as [8]:

$$|\gamma_{a}|=1-\frac{\text{sin}\theta }{\nu }\cdot \text{sec}\varphi \cdot \Delta f_{DC}$$ (6)

**Fig 2:**
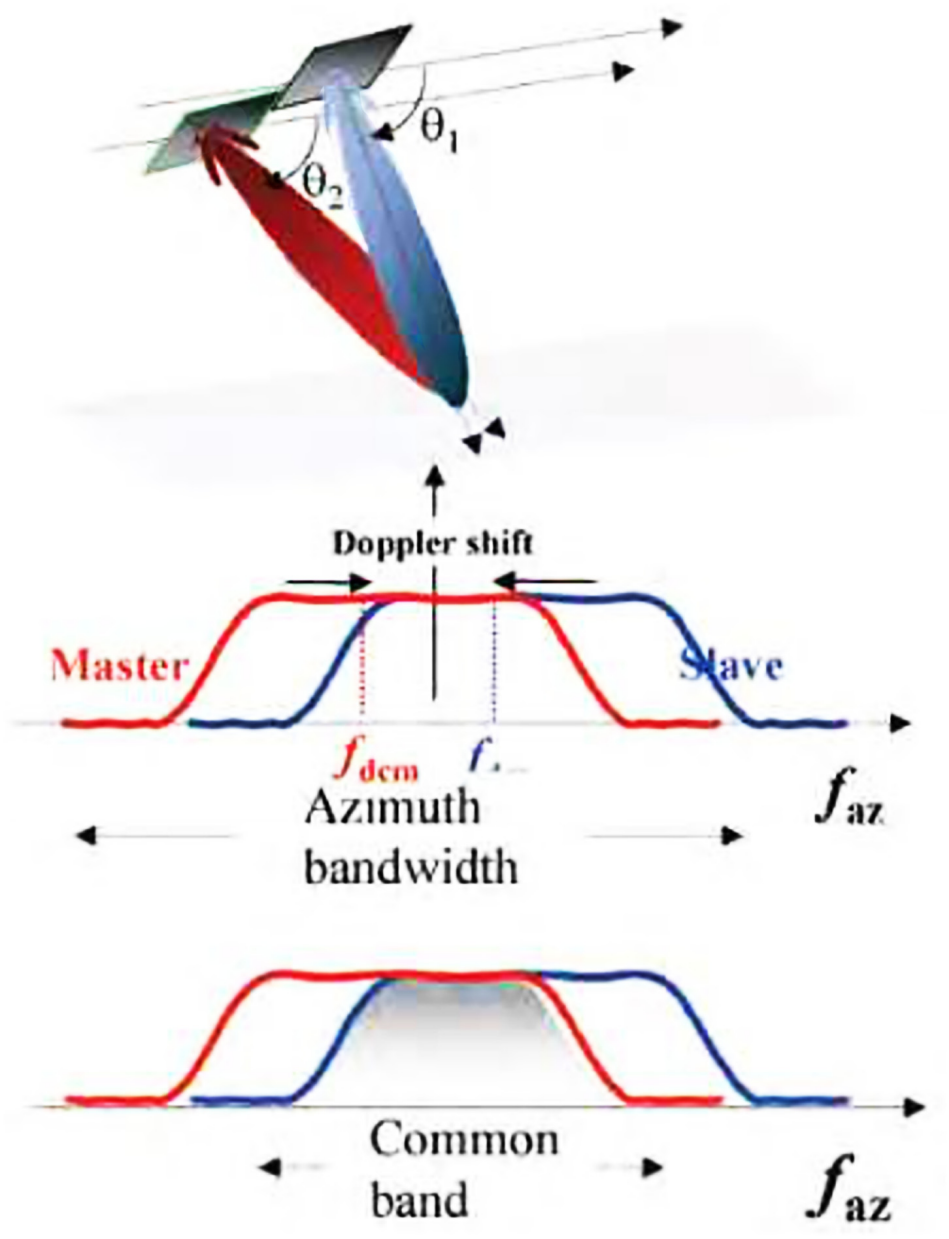
Decoherence by frequency spectrum of azimuth.

Visibly, for repeat mode different squint angles can cause inconsistency of azimuth doppler centroids, which brings the frequency spectrum offset of azimuth (see Fig 2). Only two single look complex images for interference have sufficient common frequency band on the azimuth, the higher coherence could be guaranteed. Therefore, in the process of imaging, it is need to adopt the consistent matched filter on the azimuth at the expense of certain signal-to-noise ratio (SNR) to ensure higher coherence, and then the interferogram with high quality is obtained. This is the fundamental idea of spaceborne imaging algorithm for optimizing coherence studied in this paper [9].

### (3) Temporal decorrelation [10]

Different spaceborne SAR platforms have different orbit revisit cycles, for example, ENVISAT ASAR needs 35 days, Terrasar-x needs 11 days, and ALOS satellite needs 46 days. Within the revisit cycle, ground surface may have some changes due to moisture, vegetation, atmospheric conditions, etc. Noise is likely to be added in the interferomitric phase, which must reduce the coherence and degrade the interferogram quality. As all side above, the decorrelation phenomenon is inevitable. However the effect of these factors can be reduced as good as possible by the appropriate selection for imaging time or polarization mode of two interference images. Novel imaging algorithm proposed in this paper will be described in next part, the results calculated by this method are compared with traditional in the third part, and finally some advantages are concluded in last part.

## Materials and Methods

It can be seen from the analysis above the factors which generate decorrelation phenomenon caused by the baseline and time is unavoidable. For decorrelation due to azimuth doppler centroid shift, this paper proposes the imaging algorithm that consistent matched filter is adopted in focusing process to ensure the greatest coherence so as to acquire the interferogram with high quality. Original data integration and the track information extraction should be complete firstly, because many raw data is stored according to the format of Committee on Earth Observation Satellites CEOS, including four types such as SAR header file, original data file and directory file. Header file mainly provides relevant parameters (such as orbit altitude, scene center position, etc.) of Spaceborne SAR platform [11–12]. The original data file contains some record information of header file and signal data received by SAR. Directory files describe the data storage structure in detail. Then, the azimuth doppler parameters estimation is carried out. The integrated original data are conducted on the doppler center estimation. The Correlation Doppler Estimator (CDE) [13] is used to estimate the doppler center frequency of imaging, and this method mainly uses the energy spectrum of the azimuth signal and its autocorrelation function of Fourier transform. Moreover, it is assumed that $h_{0}\left(n\right)={h^{\prime }}_{0}(nT)$ is a random process, and its can be expressed as [14].

$$R_{0}(k)=E\left\{h_{0}(k+m)h_{0}^{*}(m)\right\}$$ (7)

The power spectrum of function above can be resolved by Fourier transform as:

$$S_{0}(f)=F\left\{R_{0}(k)\right\}=\sum_{k=-\infty }^{\infty }R_{0}(k)e^{-j2\pi kTf}$$

$$\Leftrightarrow R_{0}(k)=F^{-1}\left\{S_{0}(f)\right\}=T\int_{1/2T}^{1/2T}S_{0}(f)e^{j2\pi kTf}df$$ (8)

When frequency shift occurs, then

$$S_{h}(f)=S_{0}(f-f_{DC})$$

$$\Leftrightarrow R_{h}(k)=e^{j2\pi kTf_{DC}}R_{0}(k)$$ (9)

In formula above, the original data can be estimated to get the autocorrelation function directly in the time domain [15].

$${\hat{R}}_{h}(k)=\frac{1}{N}\sum_{i=1}^{N}h(k+i)h^{*}(i)$$ (10)

The doppler centroid can be estimated by

$${\hat{f}}_{DC}=\frac{1}{2\pi kT}\text{arg}\{{\hat{R}}_{h}(k)\}$$ (11)

The quadratic-multinomial fitting is introduced to get the relation between the doppler centroid *f*_*DC*_ and slant range variation, namely,

$$f_{DC}={\hat{f}}_{DC0}+{\hat{f}}_{DC1}b+{\hat{f}}_{DC2}b^{2}$$ (12)

In $b=\text{int}\left(\frac{\rho -\rho_{0}}{\Delta r}\right)$ of formula (12), int(⋅) represents integer part reserved, Δ*r* is the sampling interval on the azimuth, *ρ* refers to the slant distance between the pixel points in azimuth and the radar. Then, these parameters of the pairs are evaluated by this method, and after consistent process doppler centroid can be expressed as:

$$\begin{array}{l} f_{DC0}=({\hat{f}}_{DC0_{1}}+{\hat{f}}_{DC0_{2}})/2\\ f_{DC1}=({\hat{f}}_{DC1_{1}}+{\hat{f}}_{DC1_{2}})/2\\ f_{DC2}=({\hat{f}}_{DC2_{1}}+{\hat{f}}_{DC2_{2}})/2\\ f_{DC}=f_{DC0}+f_{DC1}b+f_{DC2}b^{2} \end{array}$$ (13)

Radar squint angle also becomes

$$\bar{\varphi }=\left(\varphi_{1}+\varphi_{2}\right)/2$$ (14)

So Δ*φ* = 0, then make |*γ*_*a*_| maximum, the greatest coherence of master-slave images can be acquired finally. Another parameter calculated is the doppler modulation frequency which can be obtained from the track information.

$$\begin{array}{l} K_{a}=-2V_{r}{}^{2}/\lambda \rho \\ V_{r}=v\cdot \text{cos}(\varphi ) \end{array}$$ (15)

In formula (15), *V*_*r*_ represents the equivalent velocity of platform to ground targets. Because the influence is negligible for imaging quality, higher-order term due to different slant distances can be neglected in the formula [16]. When parameter estimation is completed, the Rang-Doppler method is used for focusing process. The range compression is complemented firstly, which is the pulse compression essentially. The matching filter function is:

$$\begin{array}{l} {\bar{H}}_{r}=\text{exp}\{j2\pi (-\frac{1}{2}K_{r}t^{2})\}\cdots (-\frac{T_{p}}{2}<t<\frac{T_{p}}{2})\\ K_{r}=B_{r}/T_{p} \end{array}$$ (16)

Where *B*_*r*_ and *T*_*p*_ represent the radar bandwidth and pulse time width respectively, and *K*_*r*_ is the range frequency ratio. After the pulse compression of range, azimuth migration correction needs to be conducted with the compressed data. The range migration is expressed as:

$$\Delta \rho =-\frac{\lambda f_{DC}}{2}(t-t_{c})-\frac{\lambda f_{R}}{4}{\left(t-t_{c}\right)}^{2}$$ (17)

*t*_*c*_ is the moment when the center of radar azimuth beam is sweeping the target. After the azimuth Fourier transform, fractional part in Δ*ρ* needs interpolation in the range doppler domain. The algorithm in this paper compensates for the range migration by the *sinc* interpolation function. The *sinc* function is expressed as:

$$s(x)=\sum_{i=-N/2+1}^{N/2}s(i)\text{sin}c[\pi (x-i)]=\frac{\text{sin}\pi x}{\pi }\sum_{i=-N/2+1}^{N/2}\frac{{\left(-1\right)}^{i}}{x-i}s(i)$$ (18)

8 points sinc kernel is introduced in this algorithm here, so N is equal to 8. The data after range migration correction should do the azimuth compression by using the azimuth matching filter:

$$\begin{array}{l} {\bar{H}}_{a}=\text{exp}\{j2\pi [f_{DC}t-\frac{1}{2}K_{a}t^{2}]\}\cdots (-\frac{T_{s}}{2}<t<\frac{T_{s}}{2})\\ T_{s}=L/v \end{array}$$ (19)

In formula (19), *L* is the length of the synthetic aperture, and then Fourier inverse transformation of azimuth is carried out, finally, single-look complex image of SAR is output.

### Study areas

**Fig 3:**
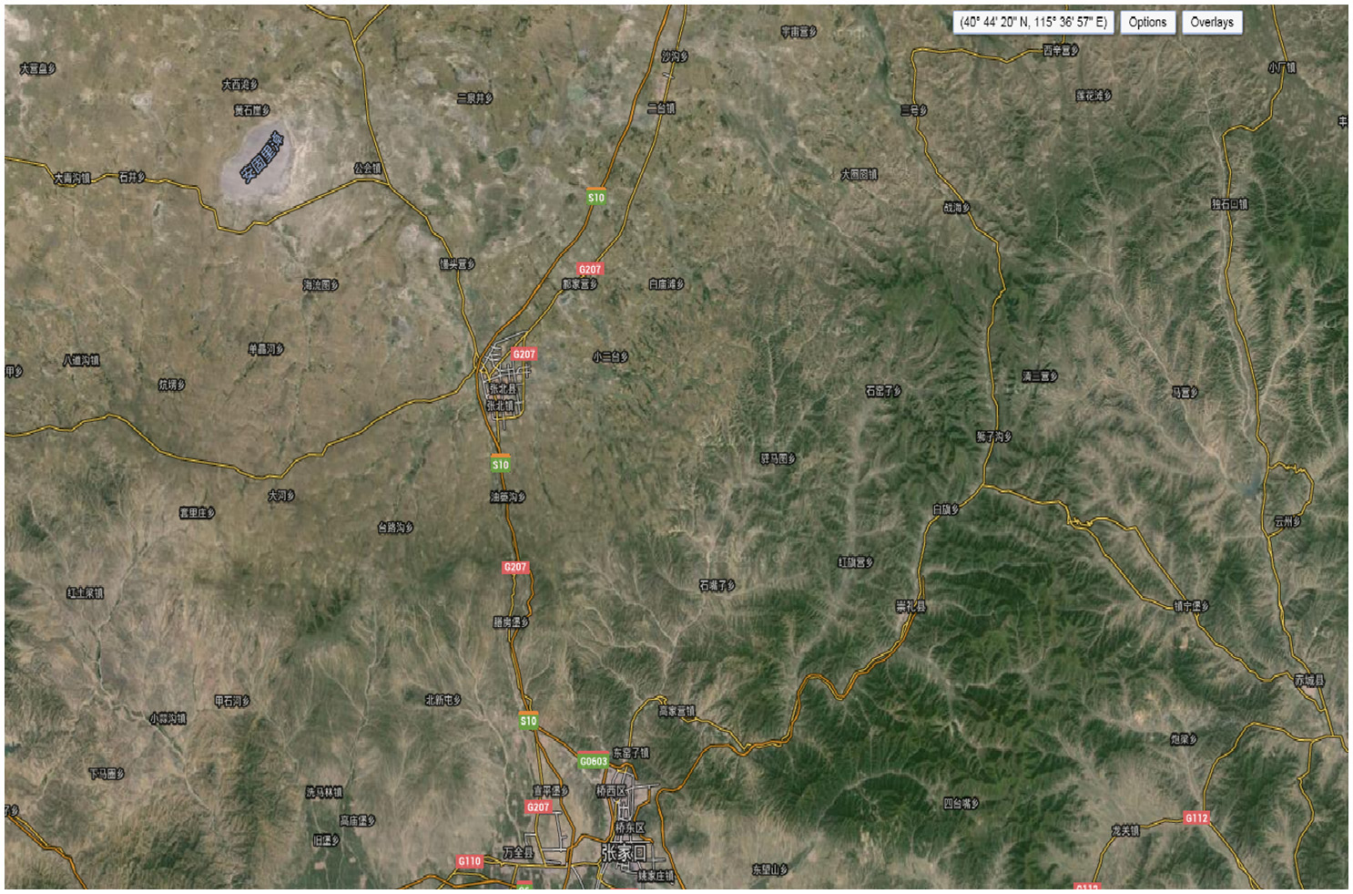
Experimental area on USGS.

Zhangbei in Hebei province, China, was selected as interest area in this paper. Fig 3 shows the satellite image of Zhangbei area from USGS. Test data are the ERS-1/2 tandem data. The imaging algorithm proposed above is used to complete the focusing process in this paper. InSAR data processing software developed by our research group for the test data was bought from European space agency (ESA). Working frequency of SAR sensor is 5.3 GHz (C wave band), and the average orbit altitude is 785 km. The parameters of radar sensor in ERS-1 and ERS-2 are completely same. They move in orbit as tandem mode, and repeat cycle is 1 day. Tandem mode greatly reduces the influence of temporal decorrelation, and improves the coherence of interference pairs, then enhances the ERS satellite interference measuring ability. ERS-1/2 Tandem level 0 RAW data and level 1 SLC data was selected elaborately for this experiment. As the header file of the level 0 RAW data contains many important parameters information for imaging, this article lists the focusing parameters of ERS-1/2 satellite platform and the key parameters of SLC data, respectively, (parameters are shown in Tables 1 and 2). In this paper, the ERS- 1/2 Tandem data of Zhangbei region was used for the experiment.

**Table 1:** Parameters for ERS 1/2.

**Wavelength*λ***	0.056565m
***K***_*r*_	41.9137×10^10^Hz/s
**PRF**	1679.878Hz
**Pulse Width**	37.10*μs*
**Sample Frequency for Range**	18.962468MHz
**Average Velocity**	7551m/s
**Antenna Length**	10m

**Table 2:** Parameters for Zhangbei SLC data.

Parameter	Master	Slave
**Platform**	ERS-1	ERS-2
**Acquired Time**	1997/10/8/03:06:47–03:07:03	1997/10/9/03:06:53–03:07:08
**Data ID**	Orbit-32585, frame = 2781	Orbit = 12912, frame = 2781
**Orbit Type**	descending	descending
**Incidence Angle**	23.231°	23.249°
**Doppler Centroid**	**449.213Hz**	**171.673Hz**
**Scene Center**	40.955N/114.625E	40.961N/114.62E
**Range Sampling Size**	7.904m	7.904m
**Azimuth Sampling Size**	3.978m	3.978m

**Fig 4:**
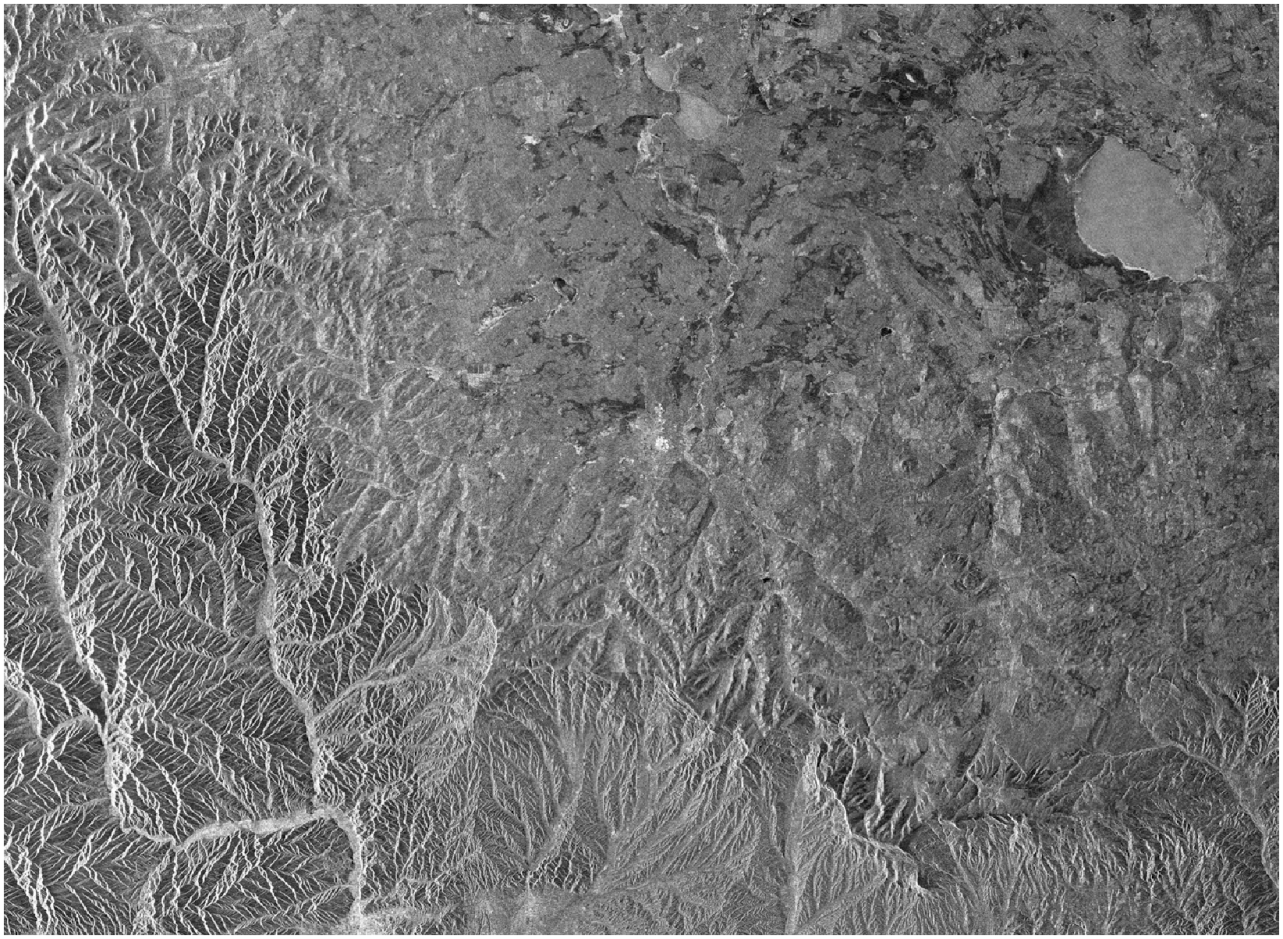
Amplitude image of SLC data after horizontal translation.

Fig 4 describes the amplitude of master SLC data in Zhangbei. Due to the two groups of data are the descending data, there is the flip-horizontal relation between the SAR amplitude images and optical images in fact. After multi-look process of azimuth with a proportion of 5:1 and master-slave images registration, the size of interested area is 3400 * 4895 (Row * Column) including 16643000 pixels totally, space baseline between two master-slave images is about 370m. Doppler center frequency deviation between pairs is about 270 Hz, accounts for about 16% of the azimuth bandwidth (i.e. PRF). Test area is located in Zhangbei region belonged to Hebei province in China. The terrain is disadvantageous for interferogram generation, mostly mountain. It can be seen from the right part of amplitude diagram in Fig 4, ups and downs of relief is more fierce, while the left part of the terrain changes more gently. It is beneficial to compare and analyze the experimental results from the imaging algorithm with the coherence proposed in this paper.

### Spectral analysis for test data

To deeply analyze the frequency spectrum information of the ERS Tandem data in Zhangbei region, it is necessary to calculate the range and azimuth power spectrum respectively according to the Level-1 SLC data provided by ground stations for comparing the spectral distribution of pairs. For analyzing the range spectrum, this paper adopted the sampling and summation method to obtain the range spectral distribution of master-slave images. Firstly, this paper sampled every 10 lines in azimuth direction, calculated their frequency spectrum energy in the frequency domain respectively next, added all obtained frequency spectrums in range direction so as to guarantee estimation of the spectral distribution correct for the whole image in the end. The same approach was applied in azimuth spectral analysis with range sampling interval of 5 rows.

**Fig 5:**
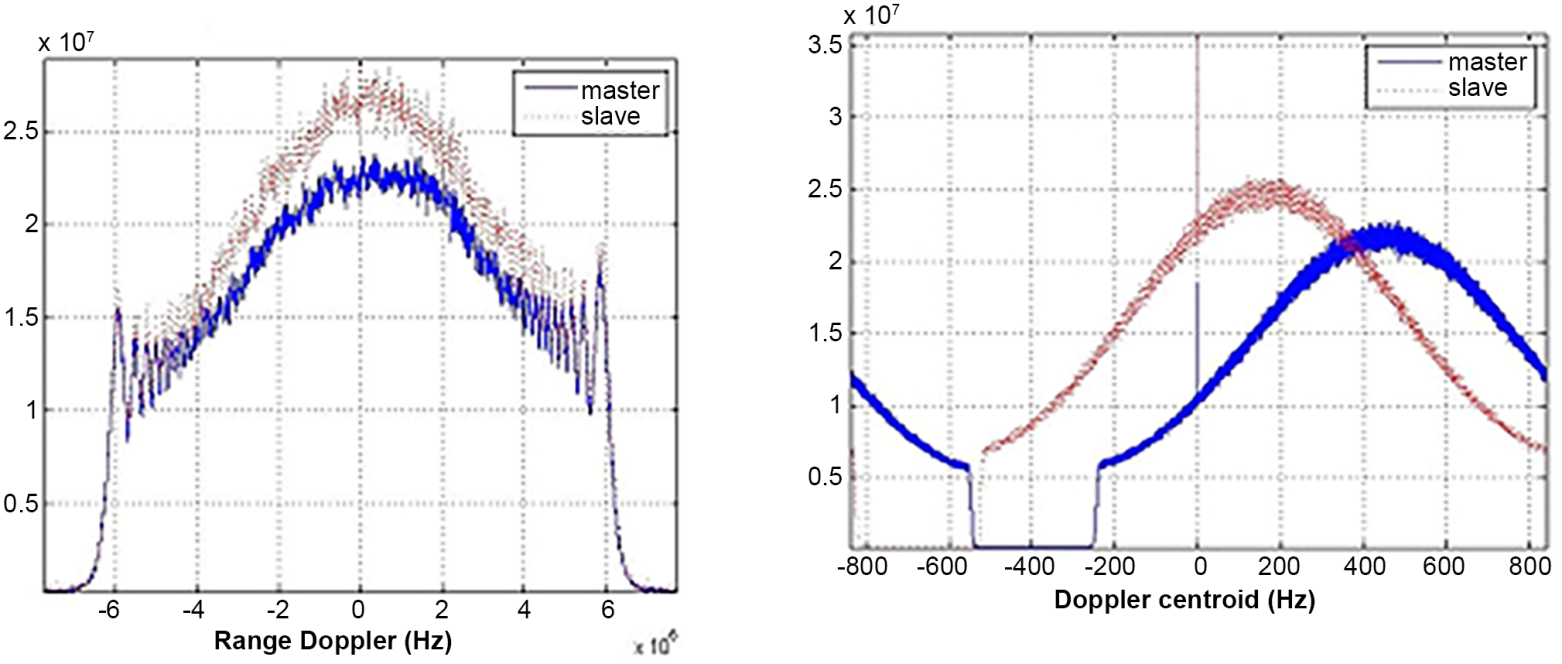
Range and azimuth frequency distribution of level 1 data.

**Fig 6:**
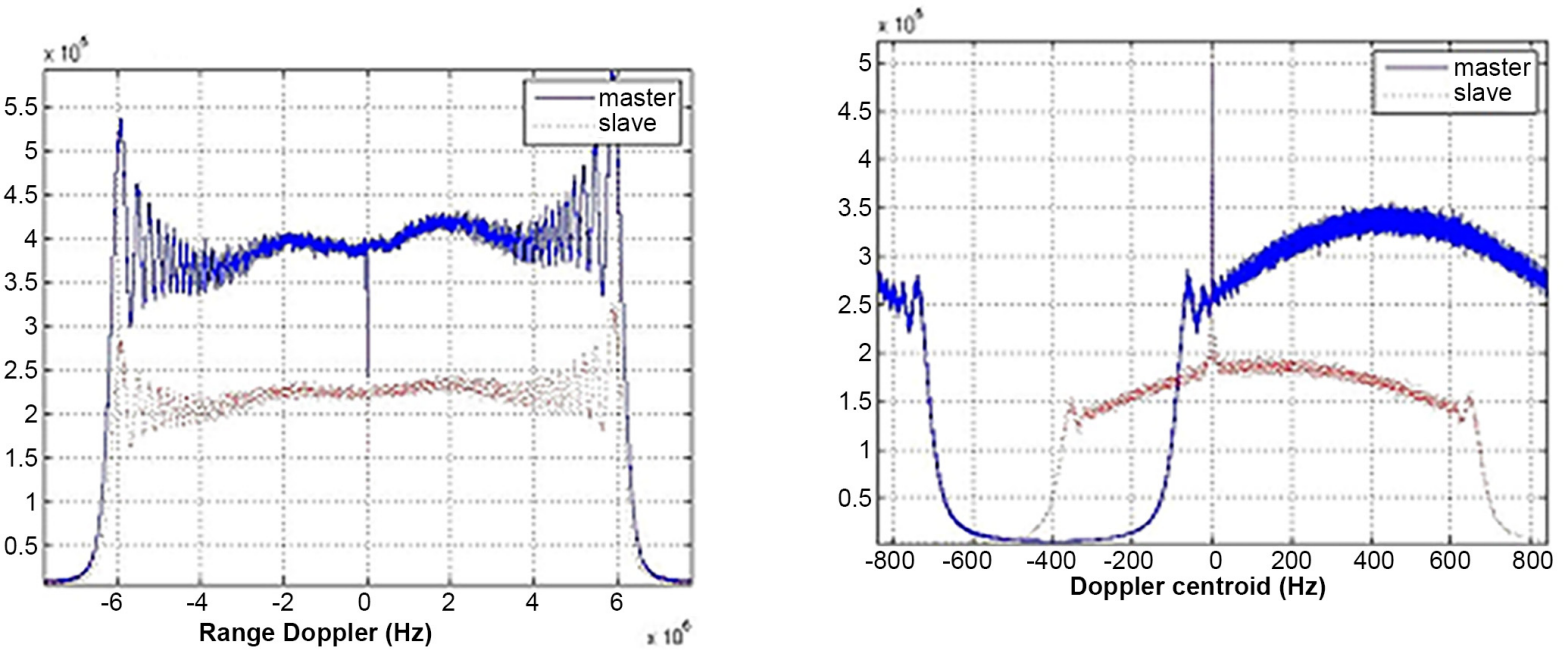
Range and azimuth frequency distribution of level 0 data before processing.

**Fig 7:**
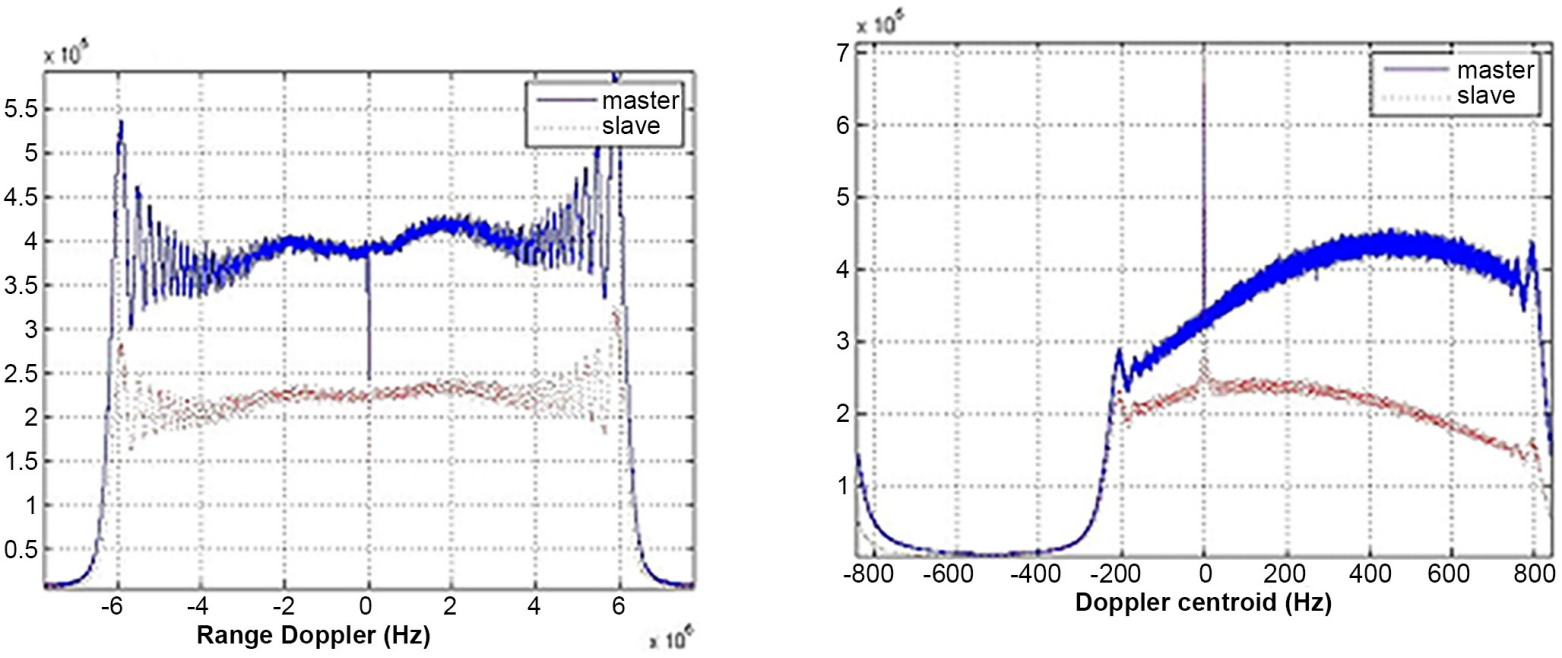
Range and azimuth frequency distribution of level 0 data after processing.

**Fig 8:**
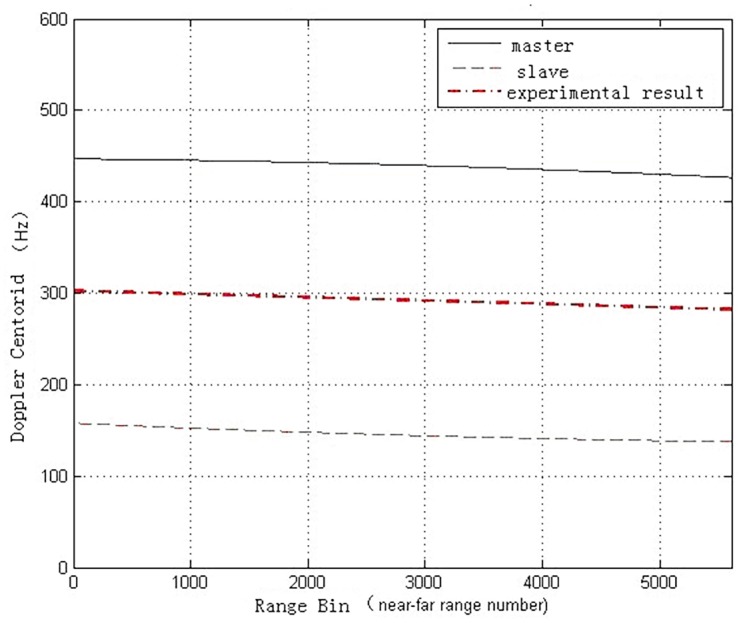
Range doppler centroid distribution after processing.

Fig 5 demonstrates the range and azimuth spectrum of Level-1 single-look complex data images focused by ground stations. It is apparent that azimuth frequency spectrums of master and slave images are inconsistent with about 270Hz gap. Moreover, in the same radar system, the range frequency spectrum is consistent with little deviation. Fig 6 shows range and azimuth spectral distribution of Level-0 data without the consistent imaging process, while Fig 7 displays the frequency spectrum of Level-0 data after coherence-optimized SAR imaging process proposed in this paper. It can be observed that there is almost no deviation in the frequency spectrum of azimuth direction between pairs, and the Doppler centroid frequency stays around 300Hz. However, the spectral shapes of Level-0 and Level-1 SAR data are only related with the weighted-window focusing parameter in range and azimuth directions. Table 3 lists the multinomial coefficient of Doppler central frequency *f*_*DC*_ estimated from Level-0 data with auto-correlation approach [17]. Fig 8 describes the distribution diagram of estimated Doppler central frequency from Level-0 SAR data in the radar range direction. Obviously, the Doppler central frequency of the pairs has received consistent processing, thus the decorrelation duto Doppler central frequency deviation in range direction between images have been reduced.

**Table 3:** Polynomial coefficients of doppler centriod for master and slave images.

Polynomial coefficients	master	slave	mean
**${\hat{f}}_{DC0}$**	446.6293	155.9330	301.28115
**${\hat{f}}_{DC1}$**	-0.0014	-0.0054	-0.0034
**${\hat{f}}_{DC2}$**	-4.5175e-007	3.7495e-007	3.84e-008
|Δ*f*_*DC*_|	295.5332(center)(Hz)		

**Fig 9:**
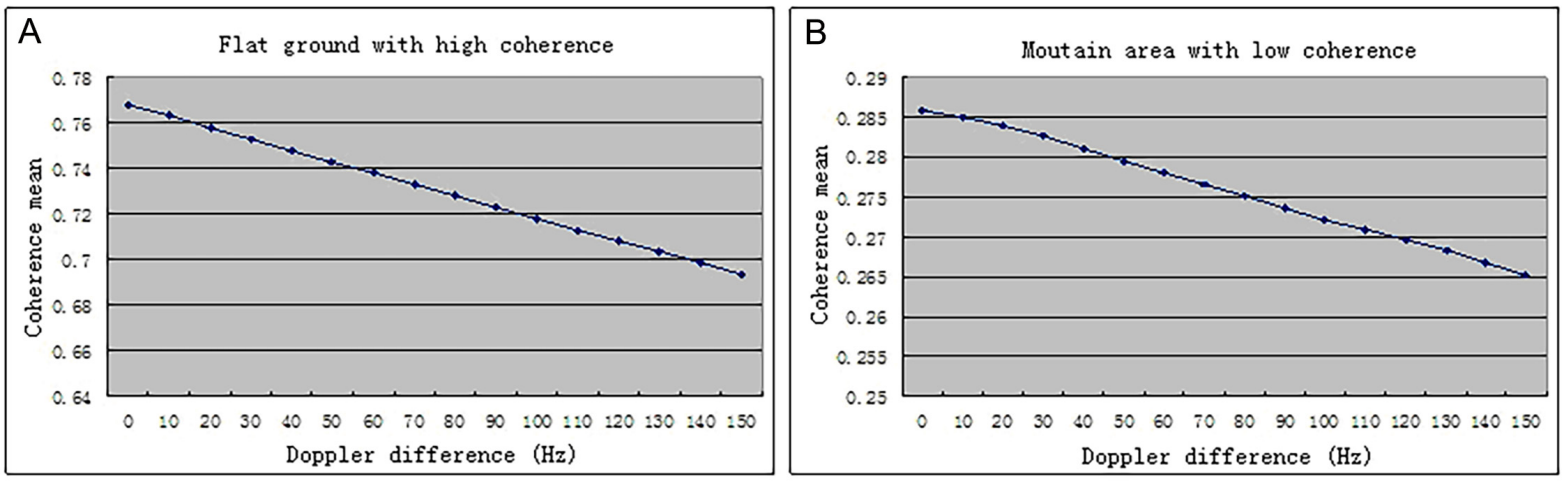
Doppler difference for flat ground and mountain area.

In order to analyze the influences of the Doppler frequency difference for correlation between master and slave images. The detailed approach is as follows: calculated the mean value of multinomial coefficients of Doppler central frequency in Table 3 as the standard at first, then changed the constant term of Doppler central frequency in images, seen the deviation of 10Hz as an interval, and plotted the coherence map of Doppler central frequency with deviation from 0Hz to 150Hz. Through the coherence map obtained from the experiment, this study selects the flat ground area with higher coherence and the mountain area with lower coherence for the statistic analysis of mean value. The detailed statistic results are shown in Fig 9. Fig 9a represents the flat ground area with higher coherence, whose coherence mean value drops from 0.76773 to 0.693472 with the deviation of 150Hz, while Fig 9b stands for the mountain area with lower coherence, whose coherence mean value drops from 0.285775 to 0.265159 with the deviation of 150Hz. It is obvious that the coherence mean value decrease with the increase of doppler central frequency deviation, changing with an approximate linear trend. When the doppler difference is 0Hz, the coherence will be the maximum, which simultaneously verifies the analysis in the third chapter about decorrelation phenomenon due to the inconsistent azimuth frequency spectrum correct.

## Results and Discussion

The Level-0 data of test area adopts the coherence-optimized imaging algorithm proposed in this paper for consistent azimuth spectrum so as to keep the maximum coherence between the pairs. However, as the Level-1 SLC data of this region are provided by the ESA ground stations, frequency deviation is approximately 270Hz, accounted for 16% of the whole azimuth bandwidth. The slave image needs to complete the registration according to master image in the beginning, then interferogram generation and coherence maps calculation finally. The two interferograms undergo identical procedure completely without the post-filtering processing. This paper analyzes experimental results on the interference fringe quality and coherence contrastively.

### Comparison and analysis

**Fig 10:**
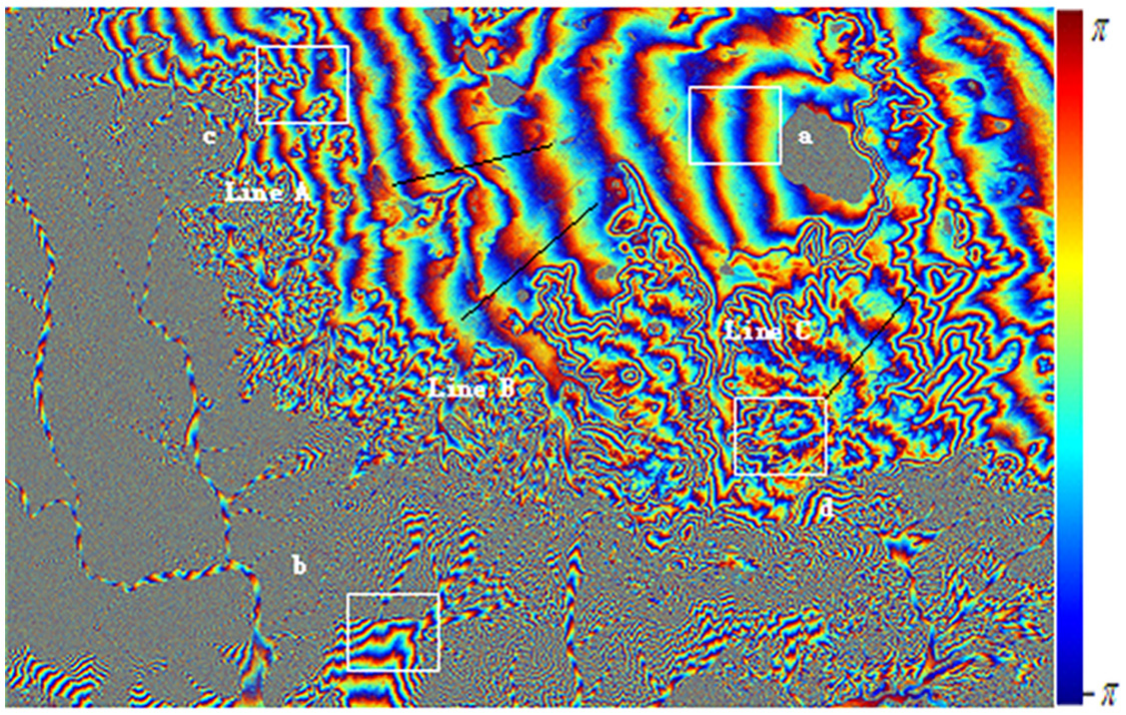
Interferogram From SLC data without coherence optimization.

**Fig 11:**
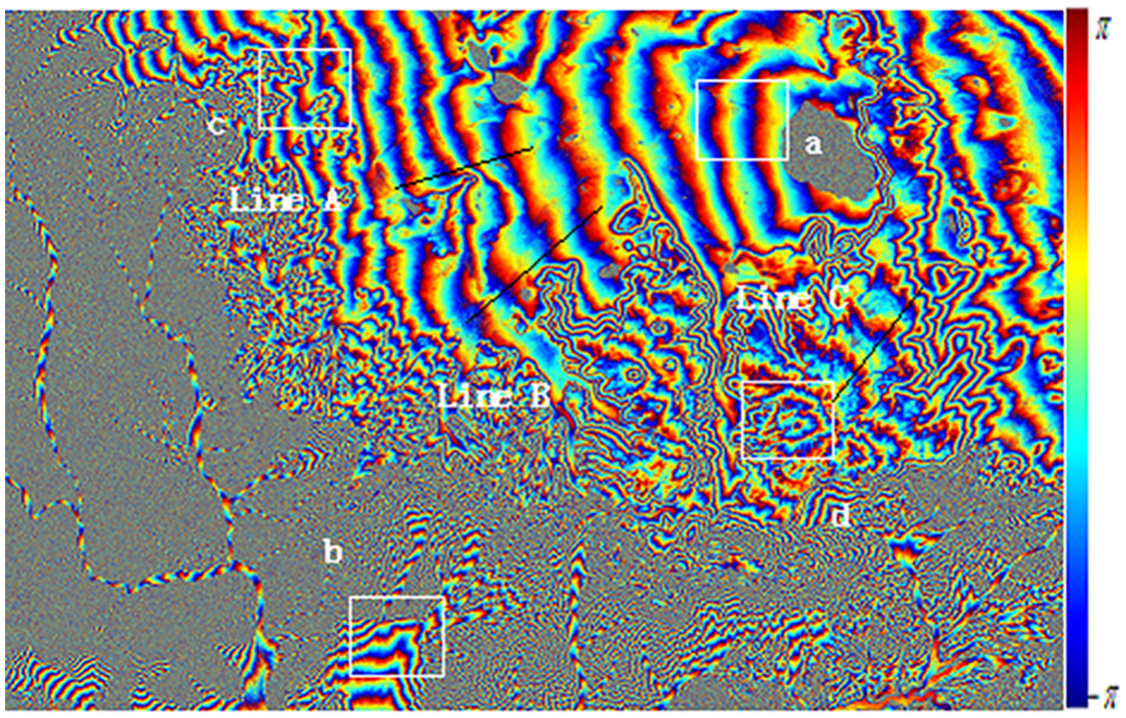
Interferogram From Level 0 Data with coherence optimization.

Figs 10 and 11 are the interference fringes of Level-1 SAR data and of Level-0 SAR data respectively. Before interferograms generation, only the Level-0 data adopted consistent imaging parameters in focusing procedure. For the sake of clearness in the recital, it is called as Level-0 below. In contrast, the SLC imaging data of Level-1 purchased from ground stations have been focused by traditional range Doppler approach, for simple, it is called as Level-1 hereinafter. After 5:1 multi-look processing in azimuth direction, the whole SAR image size is 3400*4895 including 16643000 pixels totally, and the data volume is very large. Since the data of ERS Tandem model designed for interference have high temporal coherence, there is no obvious difference in overall interference fringe. However, for quantitative analysis of the interference fringe quality, this paper cut out four fringe blocks as typical regions with the same size of 400*400. The four blocks are marked with letters a-d and plotted with white boxes respectively in Figs 10 and 11.

To evaluate and analyze interference fringe quality quantitatively, this paper applies standard deviation *σ*_*ϕ*_ [18] of phase as the evaluation parameter of fringe quality. It is defined as:

$$\sigma_{\phi }={\left(\frac{\sum_{N}{\left(\phi (i,j)-\bar{\phi }(i,j)\right)}^{2}}{N-1}\right)}^{\frac{1}{2}}$$ (20)

**Fig 12:**
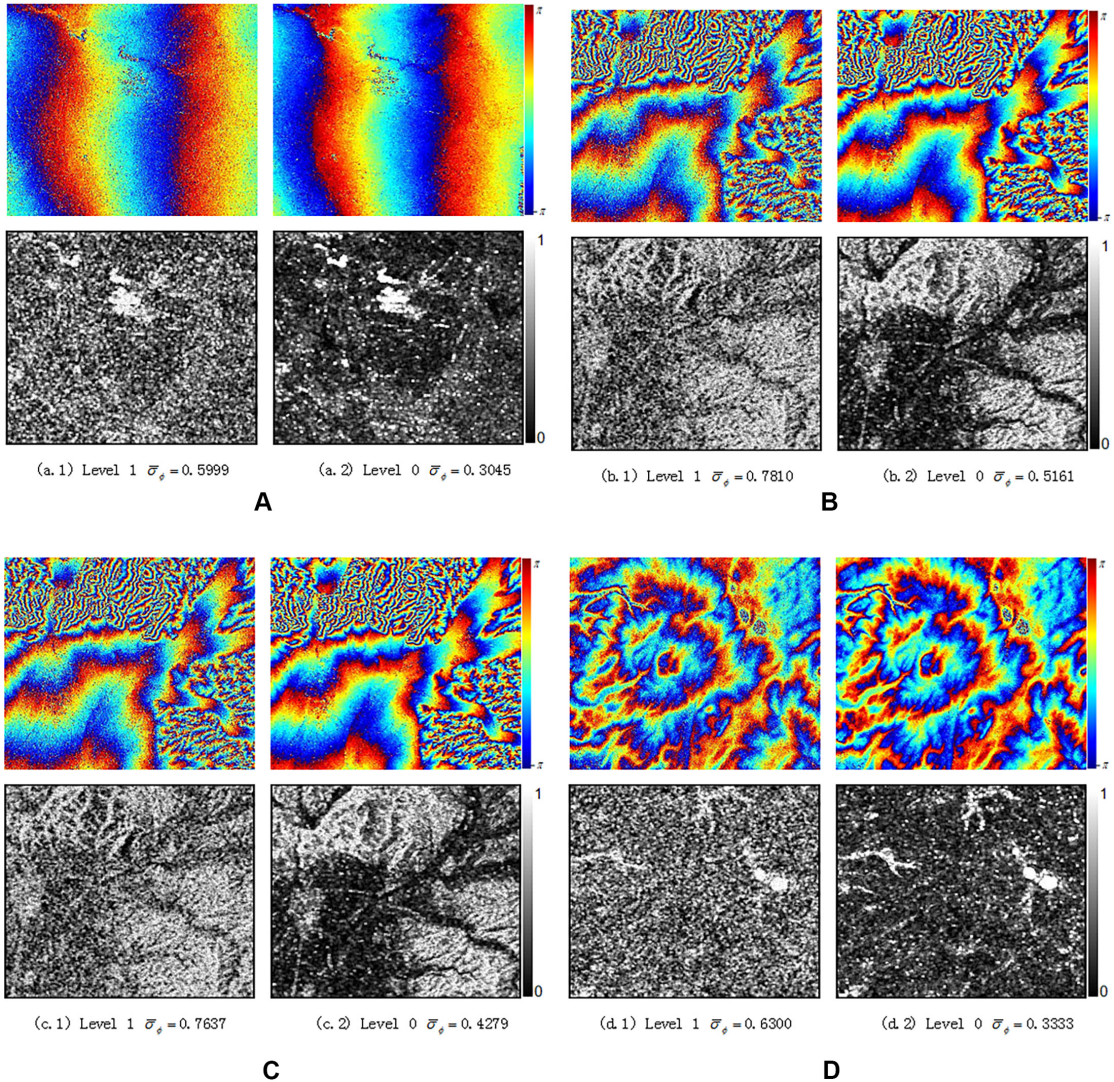
Interferogram quality comparison from SLC data (left) and level 0 data (right).

In formula (20), $\bar{\phi }(i,j)$ denotes the mean value of linear phase gradient within the rectangular window. The estimation value of $\bar{\phi }(i,j)$ is obtained by calculating the first-order derivative of phase changes (i.e. the linear changing trend of phrase) along the range and azimuth direction in the window respectively, and taking the mean value of the range and azimuth phase after removing the linear trend. The window size adopted in this paper is 5*5 and thus the number N in formula (20) is 25. The phase standard deviation is sensitive to noise amplitude of interferogram, and its value can be estimated from interferometric phase directly. Therefore, its value can be regarded as a reference index for interferometric phase quality (see Fig 12).

### Coherence statistics

As the SAR imaging algorithm proposed in this paper has fully considered the decorrelation influences caused by the inconsistent Doppler frequency spectrum in azimuth direction, it can guarantee the maximum similarity of signals in azimuth direction during the focusing procedure for the two pairs in interferometric operation so as to acquire the relative high coherence in theory. This paper calculates coherence maps of test data. Subsequently, statistic analysis for the coherence maps obtained through the imaging algorithm proposed in this paper and the coherence maps obtained from the Level-1 data products is implemented to verify the superiority of imaging algorithm in this paper.

**Fig 13:**
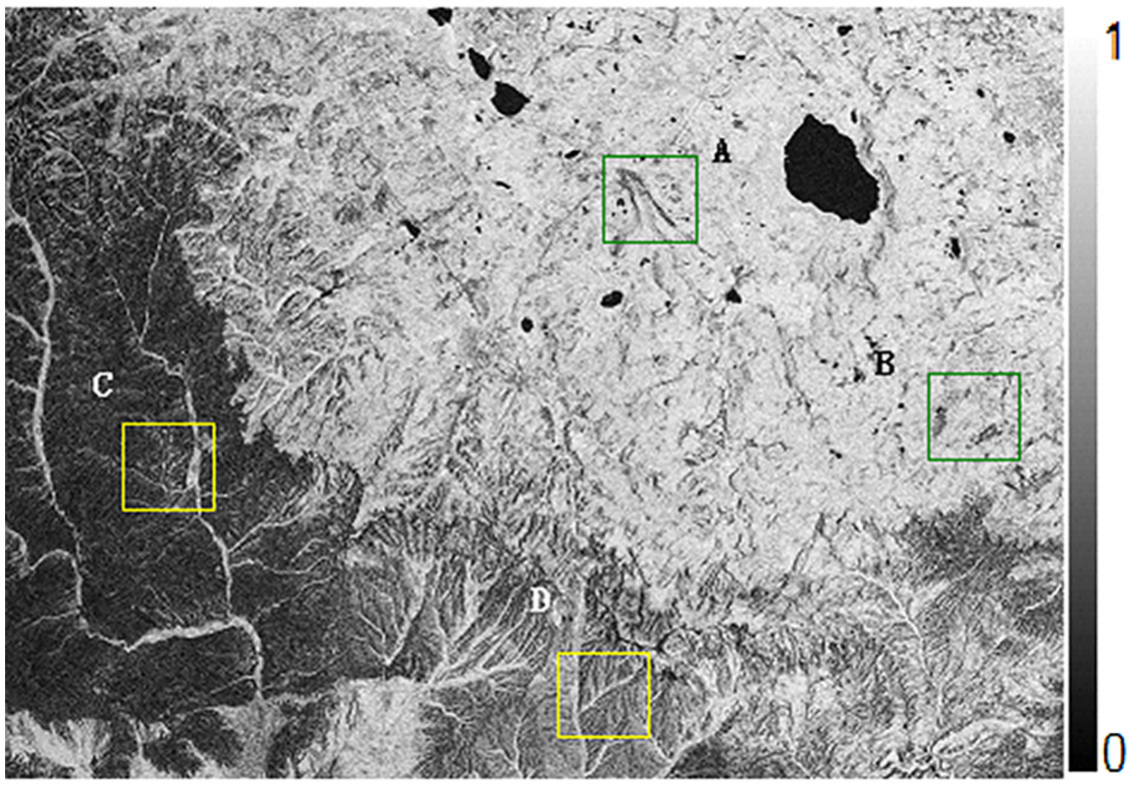
Coherence map from level 1 data without consistent doppler parameters.

**Fig 14:**
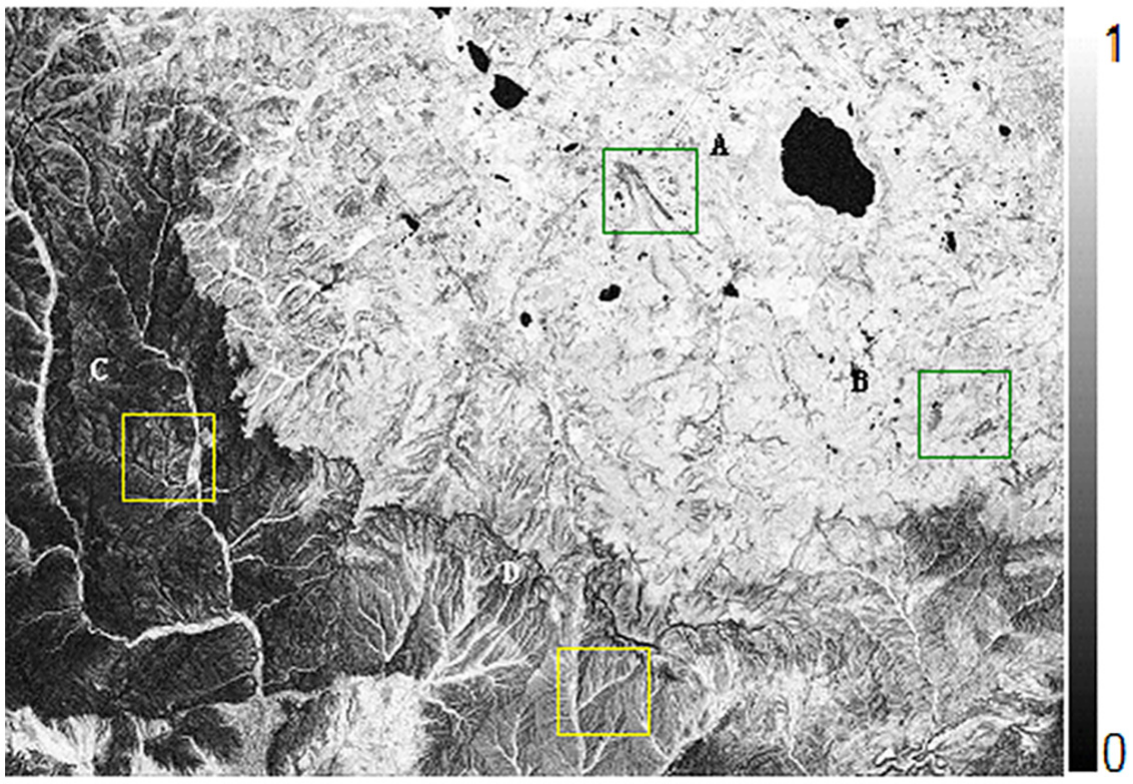
Coherence map from level 0 data with consistent doppler parameters.

**Fig 15:**
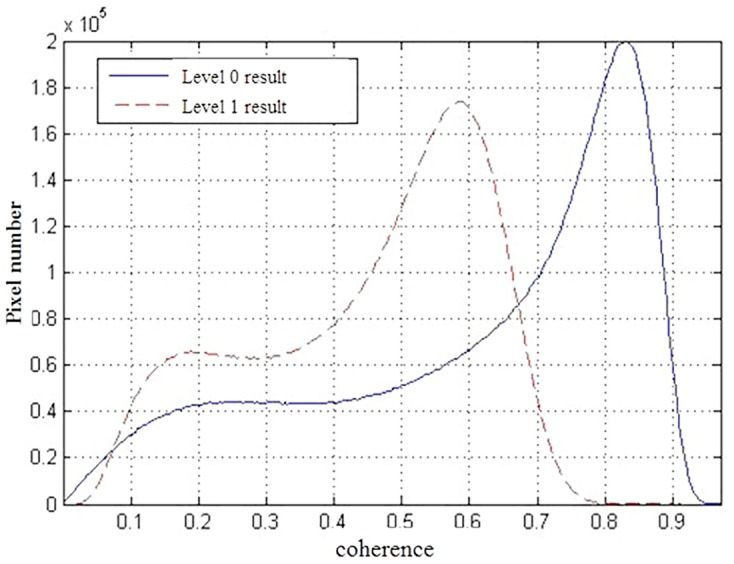
Coherence histograms of interferometric results.

Figs 13 and 14 are the coherence maps obtained from results of Level-1 and Level-0 data respectively. Obviously, it can be seen that the coherence of Level-0 results is higher than that of Level-1. Fig 15 plots the coherence histogram of two results, the coherence can be visually viewed from two histograms. The main part of histogram for Level-1 results lies in 0.4~0.7 while that of Level-0 is in 0.6~0.9. Therefore, it can be concluded that the mean value of coherence obtained from spaceborne SAR imaging algorithm proposed in this paper has been improved significantly compared with the mean value from Level-1, which increased from 0.451613 to 0.611423 (see in Table 4).

**Table 4:** Statistical values of coherence in the experiment results.

Statistical Values	Minimum	Maximum	Mean	Mean Square Error
**Level 0 Result with Proposed Method**	0.00008	0.97058	**0.61142**	0.23811
**Level 1 Experiment Result**	0.01192	0.91449	**0.45161**	0.17207

This paper chose the gentle flat ground areas A and B with higher coherence and the mountain areas C and D with lower coherence as four typical areas and they were marked out in coherence maps with blocks. By comparing the mean value of coherence in these four areas, this paper further analyzed the coherence increased ratio in different topographic regions. The ratio is defined as [18]:

η=|γ¯0||γ¯1|−1 (21)

**Fig 16:**
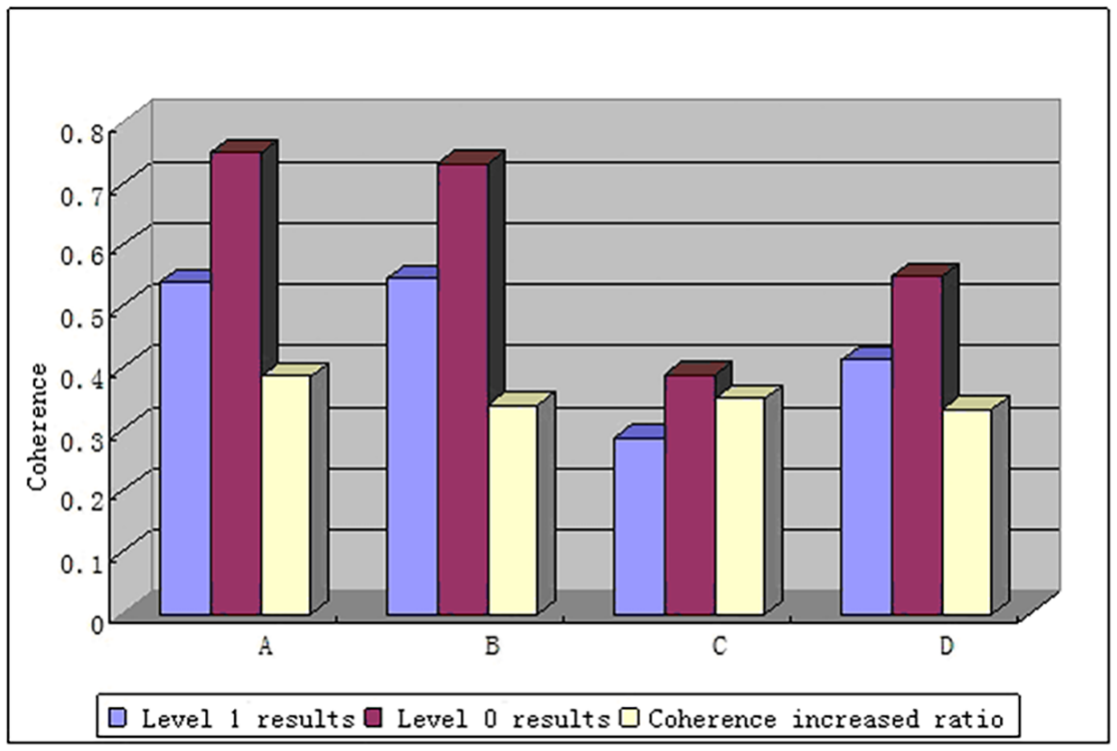
Coherence results and increased ratio for block area.

In formula (21), $\left|{\bar{\gamma }}_{0}\right|$ represents the mean coherence of chosen regions from Level 0 coherence map, while $\left|{\bar{\gamma }}_{1}\right|$ signifies the mean value of coherence from Level 1 map. In Fig 16, the mean coherence of Level-0 data increased more than 0.3 for either mountain or flat ground areas. It is also seen that the coherence of flat ground areas is slightly higher than that of mountain areas. The reason may be that the terrain fluctuation in mountain area is very large and the local incident angle of radar will change acutely in such area, which will cause frequency spectrum deviation in range direction for master and slave images greatly affected by the decorrelation due to baselines.

## Conclusions

As we know, that the coherence can be seen as an important index to evaluate the images similarity and interferogram quality [19]. The spectral consistence in azimuth direction for the two imaging pairs processed by the imaging algorithm proposed in this paper is far better than that by the traditional algorithm. The SLC imaging product does not completely serve the InSAR technology, but they endeavor to satisfy the most extensive technical needs for the maximum SNR and the optimal imaging quality. However, as a topographic mapping technology with high precision, the SAR technology is equipped with its own technical characteristics. In order to acquire the precise terrain phase information or deformation, it is necessary to guarantee the maximum coherence of received echoes between two pairs. Based on the theory of InSAR technology, this paper fixed the Doppler frequency spectrum between master and slave images in the imaging process. After azimuth focusing, two pairs possessed consistent azimuth direction can be acquired finally. This paper compared and analyzed the results of two methods in fringe quality and coherence maps. It is observed that the coherence-optimized Spaceborne SAR imaging algorithm is significantly superior to traditional Range Doppler algorithm. Coherence and phase standard deviation has fully demonstrated the superiority of the algorithm in the research and application of InSAR technology.
